# Communities of Practice and Living Labs: A Scoping Review of Principles and Methodologies for Involvement of Lived Experience Experts in Health and Healthcare Research

**DOI:** 10.1111/hex.70601

**Published:** 2026-02-22

**Authors:** Eliza Watson, Sadia Afrin, Katrina M. Long, Clarissa Giebel, Chris Moran, Darshini Ayton

**Affiliations:** ^1^ School of Public Health and Preventive Medicine Monash University Melbourne Australia; ^2^ School of Primary and Allied Health Care Monash University Frankston Australia; ^3^ National Centre for Healthy Ageing Frankston Australia; ^4^ Department of Primary Care and Mental Health University of Liverpool Liverpool UK; ^5^ NIHR Applied Research Collaboration North West Coast Liverpool UK

**Keywords:** co‐creation, codesign, community of practice, health research, lived experience, living lab, participatory design

## Abstract

**Background:**

There is growing recognition of the importance of including those with lived experience in health and healthcare research. Communities of practice and living labs are two increasingly common approaches being used for the involvement of lived experience experts.

**Objective:**

To examine and compare the key characteristics of communities of practice and living labs in health and healthcare research that include people with lived experience.

**Methods:**

A scoping review was conducted, and MEDLINE, SCOPUS and CINAHL were searched for relevant literature. One reviewer undertook title and abstract screening, two carried out full‐text screening, and one conducted data extraction, with 10% of the data extracted by a second reviewer.

**Results:**

Of the 63 included studies, 19 were focused on communities of practice and 44 on living labs. Communities of practice were mainly used for knowledge exchange, to support people living with conditions or to improve health literacy, services and policies. Living labs were mainly used for co‐design and testing of products and services, and for collaboration between academia, health services and those with lived experience.

**Conclusion:**

Both approaches are used for effective inclusion of people with lived experience and can be flexibly applied depending on the desired outcome. CoPs are typically used for creating and sharing knowledge, and living labs are used for developing technology and for large‐scale research networks.

**Patient or Public Contribution:**

Patients and members of the public were not involved in conducting this review. The broader project that this review was conducted within has a Public Advisory Committee, which includes one person living with dementia and three former carers of people living with dementia. They have provided guidance on our project as a whole. This review was conducted to inform another part of the broader project and is focused on studies that involved people with lived experience.

## 1 Introduction

Researchers and funders are increasingly prioritising involvement of those with lived experience in research projects [1, 2, 3]. Consumer and Community involvement (CCI), as it is termed in Australia, or Patient and Public Involvement (PPI) in the United Kingdom, Europe and Canada, is an approach where those with lived and living experience, including those affected by a condition, family members and unpaid caregivers (collectively referred to as lived experience throughout this article) are involved across the research process from agenda setting, study design, data collection and analysis, and dissemination, to implementation and delivery of services for end users [4, 5, 6]. Involving lived experience experts in research and service design and delivery offers opportunities for researchers to collaborate, listen to concerns and feedback, and make changes to improve the validity of the research process, better align outcomes with lived experience expert preferences and strengthen adherence and uptake [4, 5]. However, the extent to which those with lived experience are involved in these stages can differ greatly and can often be viewed as tokenistic [7].

While not strictly CCI, co‐design, creation and production (or ‘co‐approaches’) offer participatory methods that value meaningful input of people with lived experience for design, development or delivery in the healthcare sector [8, 9]. These co‐approaches offer an avenue for creating services or products that are designed ‘with’ not ‘for’ and can benefit those involved by increasing confidence, knowledge and skills [9, 10]. Researchers undertaking co‐approaches are often looking to new methods for collaboration and involvement with those with lived experience. In health settings, two approaches have become popular to address this: community of practice (CoP) and living lab.

### 1.1 CoP

CoP is a concept rooted in social learning theory, built around the idea of a collective and shared learning experience [11, 12]. Formally defined by Wenger [12, 13], CoPs are described as having practices that are created over time by the sustained pursuit of a shared enterprise. Wenger identifies three dimensions of CoPs:

1. Mutual engagement (community): Bringing together a diverse group of people to engage in the actions of the community. Engagement draws on what participants do and know, and their ability to connect meaningfully to the contributions and knowledge of others.
2. A joint enterprise (domain): A shared interest or purpose that brings the participants together and defines the community. Participants may have different beliefs and views, but they have a shared overall goal that they work towards together. The enterprise creates a focus for the community and spurs action.
3. A shared repertoire (practice): The shared resources, actions, routines, stories and ways of doing things that the community adopts as part of its practice.

The ways of describing CoPs have expanded over time by Wenger and others, but the essence of a CoP can be summarised by Wenger's 1998 work as a group of people with a shared interest who are regularly engaged towards a common goal [12, 14].

### 1.2 Living Lab

Living labs can be defined as ‘open innovation ecosystems in real‐life environments based on a systematic user co‐creation approach’ (p. 18), integrating research and innovation with end users at the centre of the process [15]. However, unlike CoPs, there is less consensus on the definition of living labs, resulting in varying definitions depending on the setting, context or aim of the research [16]. Bergvall‐Kåreborn et al. [17] discuss this complexity, noting that living labs can also be seen as a methodology, an environment, a system or an innovation approach.

Living labs were originally used in the context of information and communication technology; however, their use has evolved over time into other settings, including healthcare [15]. The European Network of Living Labs (ENoLL) identifies six key characteristics of living labs [15, 18]:

1. Active User Involvement: The active participation of stakeholders through multiple stages.
2. Multi‐Stakeholder Participation: Engaging and involving a broad range of stakeholders from government, academia and the private sector to citizens.
3. Orchestration: Living labs orchestrate connections between stakeholders to ensure innovations reach shared objectives.
4. Co‐creation: Fostering top‐down or bottom‐up design and development that brings together all relevant stakeholders.
5. Real Life Settings: Living labs operate in real‐world environments to create practical solutions.
6. Multi‐Method Approach: Methods used in living labs are chosen based on the desired outcomes and can differ depending on the project.

### 1.3 Understanding the Approaches

These definitions tell us how these two approaches *can be used*. However, it is important to explore how they are practically *being used* with people with lived experience in order to understand which approach is most appropriate to achieve a desired outcome when working with people with lived experience. This scoping review aims to examine and compare the key characteristics of CoPs and living labs that involve those with lived experience in the health and healthcare space.

## 2 Methods

### 2.1 Search Strategy

A search was conducted in February 2024 and repeated in February 2025 using the following databases: MEDLINE, SCOPUS and CINAHL. The search was repeated in February 2025 using the same search strategy, in order to capture articles published within February 2024 and February 2025 and ensure up‐to‐date references were included. Search terms and keywords were centred around three categories: ‘community of practice’, ‘living lab’ and ‘consumer and community involvement’. Terms for CCI vary by region, and as such, a variety of alternative terms were also used, including PPI, community engagement, consumer participation, lived experience, co‐design and participatory design. Medical Subject Headings (MeSH) terms were used (see Supplemental File 1). Reference lists of reviews identified during title and abstract screening were searched for additional papers. Inclusion criteria were studies in English, those with a human healthcare or health‐related focus, and studies that stated they had involved people with lived experience as more than subjects of the study (i.e., designing with, not for) [10]. Articles which used a CoP or living lab as an approach, those which described the development of a CoP or living lab, and protocols and case studies were included. Exclusion criteria were: conference abstracts, reviews, commentaries and editorials, and book chapters; studies which did not include lived experience experts; and studies which were not focused on the use or development of a CoP or LL. There were no date restrictions.

**Figure 1:**
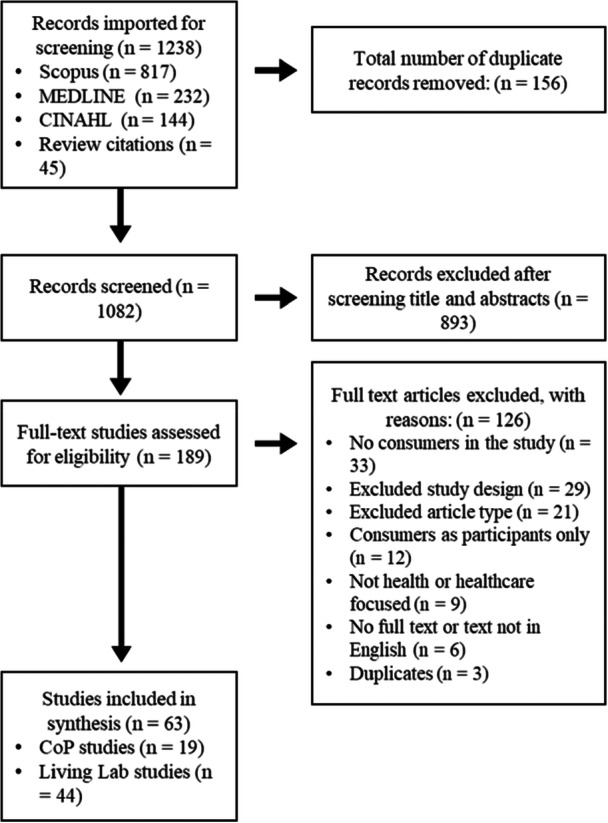
PRISMA‐ScR flow diagram illustrating the stages of the study selection process.

The search identified 1238 studies, which were imported to Covidence, and duplicates were removed, resulting in a total of 1082 articles. One researcher conducted the title and abstract screening (E.W.) and discussed any titles or abstracts that did not clearly fit in the inclusion or exclusion criteria with two of the authors (D.A. and S.A.). All full texts were screened by two authors (E.W. and either D.A. or S.A.). This resulted in 63 articles for inclusion in the review (Figure 1). One author conducted data extraction on all papers (E.W.), with 10% of papers extracted by a second researcher (S.A.). Conflicts were discussed and resolved as needed between the three researchers.

The review is reported according to the Preferred Items for Systematic Reviews Extension for Scoping Reviews (PRISMA‐ScR, see Supplemental File 2) [19]. A protocol for this review was not registered.

### 2.2 Data Extraction

Data extraction was performed in Covidence. We developed a custom extraction template to address our review questions (see Supplemental File 3). The extraction focused on understanding how each article had defined a CoP or living lab, the components or activities of a CoP or living lab that were used in the study, what additional methodology was used alongside these, the outcome of the CoP or living lab, the setting, participants and how each had involved people with lived experience. Following data extraction, content analysis was conducted using NVivo 14. Data were coded inductively to identify key characteristics of CoPs and living labs using phrases and themes from the included studies. The same broad categories were used for both CoPs and living labs; these were: framework or definition used, aim and purpose of using each approach, methodology, participant and setting. Within these categories, CoPs and living labs were coded separately.

## 3 Findings

### 3.1 Overview of Included Studies

A total of 63 articles were included in the review. Of these, 30.2% (*n* = 19) were about CoPs and 69.8% (*n* = 44) were about living labs. Some CoPs and living labs were reported in multiple articles. Two CoPs were reported in four articles, resulting in 17 unique CoPs [20, 21, 22, 23]. Six studies that were based on different datasets or case studies from the same three living labs resulted in 42 articles on unique living labs [24, 25, 26, 27, 28, 29]. A further two studies were part of the same project, but different living labs were used in each [30, 31].

Included CoP studies were mostly conducted in Europe (*n* = 8, 42%), followed by the United Kingdom (*n* = 5, 26%). Included studies were published between 2006 and 2025.

The vast majority of living lab studies were conducted in mainland Europe (*n* = 31, 70%), and more specifically in the Netherlands (*n* = 9, 21% of all included living lab studies). One study used a living lab approach to develop a CoP, which is included in Section 3.7 [32]. Included living lab studies were published between 2009 and 2025 (Table 1).

**Table 1:** Summary of study characteristics (*n* = 63).

First author	Year	Title	Country in which the study was conducted	Type of study	Aim of the study	Setting
**Community of practice studies**						
Bailey et al. [47]	2023	Vancouver's Alcohol Knowledge Exchange: Lessons Learned From Creating a Peer‐Involved Alcohol Harm Reduction Strategy in Vancouver's Downtown Eastside	Canada	CoP	To describe experiences of producing community‐led alcohol policy through the Alcohol Knowledge Exchange	Online
Conklin et al. [33]	2011	Knowledge‐to‐Action Processes in SHRTN Collaborative Communities of Practice: A Study Protocol	Canada	CoP	To increase understanding of the knowledge‐to‐action processes mobilised through CoPs that are working to improve the health of Ontario seniors	Online and in‐person (location of in‐person not defined)
Dinesen et al. [34]	2013	Attitudes of COPD Patients Towards Tele‐Rehabilitation: A Cross‐Sector Case Study	Denmark	CoP	To describe patients' attitudes towards tele‐rehabilitation in home surroundings	Online
Dinesen et al. [35]	2019	Integration of Rehabilitation Activities Into Everyday Life Through Telerehabilitation: Qualitative Study of Cardiac Patients and Their Partners	Denmark	CoP	To explore the experiences of cardiac patients and their partners who participated in the Danish Teledialog Telerehabilitation Program	Online
Ekberg et al. [36]	2010	Web 2.0 Systems Supporting Childhood Chronic Disease Management: Design Guidelines Based on Information Behaviour and Social Learning Theories	Sweden	CoP	To investigate what particular support Web 2.0 systems can give to human social learning systems, such as CoPs, in the area of chronic disease management, exemplified by childhood diabetes. And, to investigate how human information behaviour theory can be used to inform design guidelines for such systems	Online
Elsey and Lathlean [37]	2006	Using Action Research to Stimulate Organisational Change Within Health Services: Experiences From Two Community‐Based Studies	The United Kingdom	CoP	To present and critique two examples of action research used to create organisational change in healthcare	In‐person (location not defined)
Giebel et al. [22]	2023	A Community of Practice to Increase Education and Collaboration in Dementia and Ageing Research and Care: The Liverpool Dementia & Ageing Research Forum	The United Kingdom	CoP	To outline the aims, components, and evaluation of the public‐facing and engaging Liverpool Dementia & Ageing Research Forum, and to provide a blueprint for setting up similar communities of practice	Online and in‐person (at a university and other meeting locations)
Giebel et al. [23]	2025	Engaging With a Community of Practice in Dementia: Impacts on Skills, Knowledge, Networks and Accessing Support	The United Kingdom	CoP	To evaluate the experiences and impacts of engaging with the Liverpool Dementia & Ageing Research Forum by different stakeholders	Online and in‐person (at a university and other meeting locations)
Ibáñez‐Carrasco et al. [38]	2023	HIV in MOTION: A Community of Practice on Physical Rehabilitation for and by People Living With HIV and Their Allies	Canada	CoP	To describe the design, implementation and evaluation of a sustainable CoP called HIV in MOTION (HIM) to advance physical activity rehabilitation interventions with adults living with HIV, clinicians, researchers and representatives from community‐based organisations	Online
Johnson et al. [39]	2012	Conducting a Multicentre and Multinational Qualitative Study on Patient Transitions	Six European countries (Italy, the Netherlands, Poland, the United Kingdom, Spain and Sweden)	CoP	To describe the process used to create a CoP for the HANDOVER Project, examining transitions of patient care	Online and in‐person at study sites
Montali et al. [40]	2022	Mirroring, Monitoring, Modelling, Belonging, and Distancing: Psychosocial processes in an Online Support Group of Breast Cancer Patients	Italy	CoP	To investigate the psychosocial processes that characterise the experience and participation of users in an internet health forum for patients diagnosed with breast cancer	Online
Muscat et al. [41]	2023	Embedding Health Literacy Research and Best Practice Within a Socioeconomically and Culturally Diverse Health Service: A Narrative Case Study and Revised Model of Co‐Creation	Australia	CoP	To present a model for a practical and sustainable working relationship across clinical and community services focused on improving health literacy	Online
Oliver et al. [20]	2020	COproduction VALUE Creation in Healthcare Service (CO‐VALUE): An International Multicentre Protocol to Describe the Application of a Model of Value Creation for Use in Systems of Coproduced Healthcare Services and to Evaluate the Initial Feasibility, Utility and Acceptability of Associated System‐Level Value Creation Assessment Approaches	The United States	CoP	To refine a five‐part coproduction value creation model for healthcare service improvement through a three‐phase study employing principles of coproduction, a CoP and discovery learning cycles	Online and in‐person (location of in‐person not defined)
Oliver et al. [21]	2021	Initial Development of a Self‐Assessment Approach for Coproduction Value Creation by an International Community of Practice	The United States	CoP	To refine a model for value creation for use in healthcare and develop a self‐assessment guide through a CoP	Online and in‐person (location of in‐person not defined)
Perestelo‐Perez et al. [42]	2020	IC‐Health Project: Development of MOOCs to Promote Digital Health Literacy: First Results and Future Challenges	Spain	CoP	To present the objectives, main activities and results of the IC‐Health project, which aimed to develop massive open online courses (MOOCs) to improve the digital health literacy skills of EU citizens	Online and in‐person at the 14 partner sites (consisting of universities, a hospital, a government representative, a non‐governmental organisation, two small and medium‐sized companies and two European networks)
Terp et al. [43]	2016	A Room for Design: Through Participatory Design Young Adults With Schizophrenia Become Strong Collaborators	Denmark	CoP	To describe if, and how, participatory design thinking and tools can help enable participation and engagement in the development of participatory mental healthcare for young adults with schizophrenia	In‐person workshops at a hospital
Thomson et al. [44]	2013	Developing Communities of Practice to Support the Implementation of Research Into Clinical Practice	The United Kingdom	CoP	To review a case where CoPs are being developed involving researchers, practitioners and service users within a health and social research programme	In‐person at health service research centres
Tolson et al. [45]	2006	Constructing a New Approach to Developing Evidence‐Based Practice With Nurses and Older People	The United Kingdom	CoP	To use participatory research to develop a sustainable approach to enable the attainment of evidence‐based nursing care for older people across the spectrum of care environments within Scotland	Online and in‐person at a university
van Schaik et al. [46]	2022	Participatory Development of CURA, a Clinical Ethics Support Instrument for Palliative Care	The Netherlands	CoP	To reflect on and describe the process of development of a low threshold clinical ethics support instrument for caregivers working in palliative care	In‐person (location not defined)
**Living lab studies**						
Andersen et al. [69]	2018	Three Living Labs in Denmark: Challenges With Co‐Design and Implementation of Health IT	Denmark	Living lab	To analyse three large‐scale technology design projects in Danish healthcare, where co‐design and implementation activities were organised in living labs	A community setting, in patients' homes, and nursing homes
Angelini et al. [24]	2016	Senior Living Lab: An Ecological Approach to Foster Social Innovation in an Ageing Society	Switzerland	Living lab	To provide an overview of the key methodologies that are important for discussing the transdisciplinary approach and the role of technologies in a living lab for older adults	A research platform
Archibald et al. [59]	2024	A Living Lab for Family Centered Knowledge Exchange in Pediatric Rehabilitation and Development Research: A Study Protocol	Canada	Living lab	To co‐design a virtual living lab with families and clinicians to support family‐centred knowledge exchange in neurodiversity and rehabilitation research	Online platform
Bamidis et al. [48]	2020	From E‐Homes to Living Labs: Founding and Organising the Greek Active and Healthy Ageing Living Lab (Thess‐AHALL) and Its Networked Services	Greece	Living lab	To revisit the development of Thessaloniki Active and Healthy Ageing Living Lab (Thess‐AHALL) and provide the key constituents for its facilitation and expansion	Senior homes, nursing homes and day care centres
Bergvall‐Kåreborn et al. [54]	2010	Participation in Living Lab: Designing Systems With Users	Sweden	Living lab	To report on the early stages of a living lab project (MyHealth@Age) and link it to the broader field of participatory design	Not defined
Blain et al. [49]	2014	Living Lab Falls‐MACVIA‐LR: The falls Prevention Initiative of the European Innovation Partnership on Active and Healthy Ageing (EIP on AHA) in Languedoc‐Roussillon	France	Living lab	To describe the MACVIA‐LR falls initiative, a cross‐cutting living lab based on the EIP on AHA at the regional level	A network of healthcare facilities and institutes, social welfare centres, and public institutions.
Bolster et al. [73]	2021	Using a Co‐design Approach to Create Tools to Facilitate Physical Activity in Children With Physical Disabilities	The Netherlands	Living lab	To describe the insights generated during co‐design related to facilitating physical activity. And, to describe the prototypes designed during co‐design, based on knowledge from evidence and the generated insights during this method, to facilitate physical activity in everyday life settings of children with physical disabilities	In peadiatric physical therapist practices
Brankaert et al. [50]	2013	Setting Up a Living Lab for Innovation in the Dementia Care Chain, a Case Study of the PhysiCAL	The Netherlands	Living lab	To present a living lab approach for design and innovation of products and services for people living with dementia.	Participant homes
Brankaert et al. [55]	2015	Innovate Dementia: The Development of a Living Lab Protocol to Evaluate Interventions in Context	The Netherlands	Living lab	To reflect living lab cases for dementia to understand how a living lab protocol can be designed that allows for safe involvement of people living with dementia in context	Participant homes
Brankaert et al. [60]	2017	The Design‐Driven Living Lab: A New Approach to Exploring Solutions to Complex Societal Challenges	The Netherlands	Living lab	To introduce the design‐driven living lab and to investigate its potential to explore innovation for dementia care challenges	Care homes and dementia day programme facility
Bygholm et al. [31]	2014	Learning From an Ambient Assisted Living Lab: The Case of the Intelligent Bed	Denmark	Living lab	To extract methodological lessons learned from an ambient assisted living lab exploring the use of intelligent beds in a nursing home	Nursing home
Bygholm et al. [30]	2017	This Is Not Participatory Design—A Critical Analysis of Eight Living Laboratories	Denmark	Living lab	To analyse eight LLs using the six guiding participatory design principles to reveal the role and participation of the healthcare professionals and the elderly	Nursing homes
Chevalier et al. [61]	2018	Home‐Based Adapted Physical Activity by Means of a Motivational Aide Solution	France	Living lab	To encourage older people to engage in regular physical activity within their own homes to maintain good health	Not defined
Ding et al. [77]	2023	User‐Developer Interaction in a Living Lab: A Case Study of an Exercise Support System for the Elderly	Japan	Living lab	To investigate the development process of an exercise support system for the elderly	Nurse station (an office for community‐based nursing staff)
Dufour et al. [56]	2023	Research Protocol of the Laval‐ROSA Transilab: A Living Lab on Transitions for People Living With Dementia	Canada	Living lab	To present the protocol of the Laval‐ROSA Transilab (a living lab aimed at improving care transitions between settings) and an overview of its processes	Not defined (collaboration between health service and research organisation but setting is not defined)
Fotis et al. [51]	2022	Co‐Creation in a Digital Health Living Lab: A Case Study	The United Kingdom	Living lab	To present the establishment of a community‐based Digital Health Living Lab, aimed at helping develop strategies in self‐managed care for older adults	Retirement housing
Gimenez et al. [52]	2024	Living Labs for Migrant Health Research: The Challenge of Cocreating Research With Migrant Population and Policy Makers	Canada	Living lab	To describe the creation of a health research living lab for the development of cocreation strategies with migrant populations, professionals and public stakeholders to address the health challenges affecting these vulnerable groups	A research institution
Julius et al. [81]	2023	Living Labs Are the Silver Lining for Creating Sustainable Health and Care for the Future	Denmark	Living lab	To present the Public Intelligence LL methodology, which facilitates sharing knowledge between sectors, domains and cultural differences	A ‘living arena’ and a virtual nursing home
Kang et al. [78]	2012	Initiation of the Suan‐Lien Living Lab—A living Lab With an Elderly Welfare Focus	Taiwan	Living lab	To describe the development of the Suan‐Lien Living Lab, a platform for multi‐disciplinary elderly product and service innovation.	Elderly care centre
Kanstrup et al. [70]	2017	Living in the Lab: An Analysis of the Work in Eight Living Laboratories Set Up in Care Homes for Technology Innovation	Denmark	Living lab	To report on a study of eight living laboratories set up in care homes for innovation in health technologies	Care homes
Leinen et al. [53]	2025	Living Lab Dementia: Process Evaluation of an Academic‐Practice Partnership in German Long Term Care for People Living With Dementia—Study Protocol.	Germany	Living lab	To describe the implementation of the Living Lab Dementia intervention and conduct a process evaluation	Care organisations and a university research institute
Michel et al. [62]	2021	What is a ‘Good Life’: Protocol for a Qualitative Study to Explore the Viewpoint of Older Persons	France	Living lab	To define the conditions for a ‘good life’ with older persons, and to create the conditions for healthcare and service providers to appropriate this knowledge in order to adjust their offer of services to the real needs of older persons	The accommodation environment of the participant
Monguet et al. [82]	2011	eHealth Living Lab Micro Innovation Strategy: A Case Study of Prototypes Through Co‐Creation	Spain	Living lab	To present a living lab co‐creation strategy to find better ways to produce, test and market eHealth systems	Hybrid—online and physical activities (setting of physical activities not defined)
Moumtzi et al. [63]	2009	Utilizing Living Labs Approach for the Validation of Services for the Assisting Living of Elderly People	The United Kingdom	Living lab	To propose a methodology approach for the validation of services for the assisting living of elderly people utilising the living labs approach for the evaluation of services	Daily living environments of participants
Noublanche et al. [27]	2019	The Development of Gerontechnology for Hospitalized Frail Elderly People: The ALLEGRO Hospital‐Based Geriatric Living Lab	France	Living lab	To propose an operational solution to promote communication between gerontechnology developers and hospitalised frail elderly patients	Geriatric department of a hospital
Noublanche et al. [26]	2020	Adapting Gerontechnological Development to Hospitalized Frail Older People: Implementation of the ALLEGRO Hospital‐Based Geriatric Living Lab	France	Living lab	To create and implement a hospital‐based geriatric living lab to promote open hospital innovation and bridge the gap between gerontechnology developers and hospitalised frail older patients	Geriatric department of a hospital
Ogonowski et al. [74]	2016	ICT‐Based Fall Prevention System for Older Adults: Qualitative Results From a Long‐Term Field Study	Germany	Living lab	To investigate participants' use of an Information and communication technology (ICT)‐based falls prevention system	Participant homes
Palmer et al. [71]	2023	A Co‐Design Living Labs Philosophy of Practice for End‐to‐End Research Design to Translation With People With Lived‐Experience of Mental Ill‐Health and Carer/Family and Kinship Groups	Australia	Living lab	To describe the implementation, evolution and transformation of the Co‐Design Living Labs program	A university‐based network of living labs
Pedell et al. [79]	2017	Methods for Supporting Older Users in Communicating Their Emotions at Different Phases of a Living Lab Project	Australia	Living lab	To understand what methods cater for the goals and emotions of older adults in a co‐design process to develop innovative solutions	Participant homes
Pigot et al. [64]	2015	Living Labs for Designing Assistive Technologies	Canada	Living lab	(1) To present why design and evaluation of assistive technologies require living labs to develop them, (2) to classify living labs in categories related to participatory design, and (3) to draw the pros and cons of living labs categories for developing assistive technologies	A ‘smart apartment’, a living lab in an alternative housing unit, participant residences
Pino et al. [57]	2013	Contribution of the Living Lab Approach to the Development, Assessment and Provision of Assistive Technologies for Supporting Older Adults With Cognitive Disorders	France	Living lab	To present the LUSAGE LL, which is engaged in the design, assessment and deployment of assistive technology solutions for older adults with cognitive impairment	Hospital departments, adult day‐care centres, users' own environment
Riva‐Mossman et al. [25]	2016	The Senior Living Lab: An Example of Nursing Leadership	Switzerland	Living lab	To discuss the challenges and the growing opportunity for nurses to assume a leading role in interdisciplinary research projects engendering social innovation	A social laboratory in a university setting
Sanin et al. [65]	2021	Creative Wellbeing. Prototyping an Arts‐Health Practice Program for Mental Health Recovery	Australia	Living lab	To develop the Creative Wellbeing Program (CWP), an initiative to deliver art activities for a hospital mental health inpatient service. And, to create a series of tools and guidelines that would help staff to run an ‘arts‐health practice’ programme	A hospital mental health inpatient service
Santa et al. [66]	2024	Facilitating Access to Mental Health Services: A Stakeholder‐Driven Improvement of the Children and Young People (CYP) as One Referral Platform	The United Kingdom	Living lab	To facilitate access by children and young people (CYP) to relevant mental health services by improving the CYP as One platform	Online
Swinkels et al. [28]	2018	Lessons Learned From a Living Lab on the Broad Adoption of eHealth in Primary Health Care	The Netherlands	Living lab	To describe the process of adoption of eHealth in primary care from the perspectives of different stakeholders	Primary healthcare centres
Swist et al. [32]	2022	Guiding, Sustaining and Growing the Public Involvement of Young People in an Adolescent Health Research Community of Practice	Australia	Living lab	To establish a way for young people to inform and guide adolescent health research and translation	In‐person and online (setting of in‐person not defined)
Tessarolo et al. [72]	2024	Developing Ambient Assisted Living Technologies Exploiting Potential of User‐Centred Co‐Creation and Agile Methodology: The CAPTAIN Project Experience	Italy/Europe	Living lab	To present the Agile approach adopted by the CAPTAIN project for developing advanced assistive technologies in the health and well‐being domain, and to evaluate the perceived effectiveness of the development process by both the developing team and the stakeholders involved in the co‐creation and testing	Pilot sites across Europe (setting of sites not defined)
Thiesen Winthereik et al. [80]	2009	Living Labs as a Methodological Approach to Universal Access in Senior Design	Denmark	Living lab	To discuss the potential of using the living lab methodology as an approach to ensuring universal access when designing for senior citizens	Nursing home
Toso et al. [67]	2023	Reflecting on Living Labs as Multi‐Stakeholder Collaborative Networks to Evaluate Technological Products for People Living With Dementia	The Netherlands	Living lab	To set up a living lab project to evaluate and improve the design of technological products and services on the market for people living with dementia	Participant homes
Vallentin‐Holbech et al. [76]	2020	Co‐Creating a Virtual Alcohol Prevention Simulation With Young People	Denmark	Living lab	To investigate how young people perceived their participation and influence in a co‐creation process that involved multiple stakeholders developing a gamified virtual reality (VR) simulation targeted at adolescent users	A high school
Van Den Kieboom et al. [75]	2019	User‐Driven Living Lab for Assistive Technology to Support People With Dementia Living at Home: Protocol for Developing Co‐Creation‐Based Innovations	The Netherlands	Living lab	To identify, longitudinally, the changing needs of people with dementia living at home and their informal caregivers by developing co‐creation‐based innovations	Participant homes
Verbeek et al. [68]	2020	The Living Lab in Ageing and Long‐Term Care: A Sustainable Model for Translational Research Improving Quality of Life, Quality of Care and Quality of Work	The Netherlands	Living lab	To describe the Living Lab in Ageing and Long Term Care, including the key working mechanisms of the interdisciplinary collaboration, highlighting the scientific and societal impact	A network covering long‐term care facilities and professional home care
Vermeulen et al. [29]	2015	eLabEL: Technology‐Supported Living Labs in Primary Care	The Netherlands	Living lab	To describe the eLabEL project, which aims to contribute to a solution of ehealth technologies in primary care through interdisciplinary collaboration with patients for selection, integration, implementation and evaluation of technologies	Primary healthcare centres
Wargnier et al. [58]	2016	Virtual Promenade: A New Serious Game for the Rehabilitation of Older Adults With Post‐Fall Syndrome	France	Living lab	To design and test a novel rehabilitation tool for older adults with post‐fall syndrome	Geriatric acute or subacute care unit

### 3.2 CoP

CoPs were mainly described based on Wenger's definition as a group of people, a social network, or an informal or organisational grouping of peers, professionals, novices or experts who share a concern or passion for something they do, and who exchange, share or deepen their knowledge and understanding through social engagement and regular interactions [21, 22, 23, 33, 34, 35, 36, 37, 38, 39, 40, 41, 42, 43, 44, 45, 46]. Ten different articles or books for which Wenger was an author were referenced, with three of those publications referenced most frequently [11, 12, 13]. Some studies had slight variations in their references to Wenger's work. For example, one protocol paper did not include a description based on Wenger's work, but a subsequent publication resulting from the protocol did reference Wenger [20, 21]. Bailey et al. [47] did not explicitly use the Wenger definition for a CoP, but described a methodology which aligned with this definition. Conklin et al. [33], while acknowledging Wenger's model for CoPs, note that the operation of CoPs in their sector extends from this model to include the linkage of frontline practice with research evidence and an understanding that the CoP is operating in the context of a knowledge network that could impact its performance.

### 3.3 Aim of CoP Studies

The topics covered in the included CoP studies broadly fell into two groups. The first was studies which looked at support and rehabilitation for those living with chronic diseases, including cancer, and infections [34, 35, 36, 38, 40]. These studies aimed to provide active rehabilitation or support for rehabilitation involving people living with an illness, healthcare professionals and care partners. These included a tele‐rehabilitation programme for patients with chronic obstructive pulmonary disease or cardiac conditions, and exercise support for people living with HIV [34, 35, 38]. Two CoPs described were web‐based support systems or forums exclusively for people living with an illness [36, 40].

The second group was studies looking at improvement of health literacy, services and policies [20, 21, 22, 23, 33, 37, 39, 41, 42, 43, 44, 45, 46, 47]. The CoPs were used as networks for the transfer of knowledge about research and services [22, 23, 33, 44], and for the co‐production of courses and materials to improve health literacy and resources [41, 42, 45]. This category included using CoPs in the development of harm‐reduction‐informed alcohol policy, value creation in healthcare settings, a quality assurance plan for patient transition, improvement of local health services for older people, and a clinical ethics support system [20, 21, 37, 39, 46, 47]. All of the studies in this second category involved both professionals, for example, healthcare workers, researchers or designers, and lived experience experts.

### 3.4 Purpose of Using a CoP Approach

The rationale for adopting CoPs across these varied topics was the ability to provide a method for knowledge exchange [20, 21, 22, 23, 33, 34, 35, 37, 38, 39, 40, 41, 42, 44, 46, 47], knowledge to action and empowerment through knowledge [22, 23, 33, 38, 39, 40, 43, 44, 45]. They also enabled network building, opportunities for connection over time, and provided a way to bring people with lived experience together with and without professionals with a common purpose to create more lasting connections than would be achieved through one‐off workshops or meetings [22, 23, 36, 38, 39, 40, 41, 44, 47].

### 3.5 Settings and Methodologies of Communities of Practice

The CoP studies mainly took place online [34, 35, 36, 38, 40, 41, 47] or a combination of online and in‐person [20, 21, 22, 23, 33, 39, 42, 45]. Fewer took place exclusively in‐person [37, 43, 44, 46]. The setting of in‐person activities was rarely stated.

Interpretations of the format and methodologies of a CoP differed across studies. Several studies reported using participatory design or action research frameworks with a CoP forming part or all of the methodology [36, 37, 45, 46, 47]. One study initially used participatory design methods, and it was only through evaluation of the design process that it was revealed that a CoP model had organically occurred amongst participants [43].

For those studies which had developed or were developing a CoP, the preferred methodologies used to create a shared repertoire for mutual engagement included web‐based platforms or portals which house resources, access to events and a way for participants to communicate [20, 34, 35, 36, 38, 41, 44], opportunities for group discussions through meetings or forums [20, 22, 23, 37, 39, 40, 41, 46, 47], and seminars/webinars, workshops and conferences held either online or in‐person where possible [20, 22, 23, 33, 38, 41, 43, 44, 45]. The frequency of these activities was not always included.

### 3.6 Involvement of Lived Experience Experts in Communities of Practice

The makeup and number of participants in the CoPs were not always stated. In those where the number was stated and the CoP included people with lived experience as well as other members, people with lived experience ranged from approximately 1% to 46% of participants [34, 38, 43, 45, 47]. Total number of participants in the CoPs (including those not with lived experience) ranged from 17 to 4000, with a median of 48 [22, 34, 35, 37, 38, 40, 41, 42, 43, 44, 45, 46, 47]. Those held in person tended to have fewer participants [37, 43, 44, 46]. As this review included protocols, the number of participants was not always available.

The type and frequency of activities or meetings within the CoPs varied across the included studies. For some studies, the activities involved meetings or seminars as the only form of interaction. van Schaik et al. [46] described a CoP that consisted of four 3‐h sessions at 6‐month intervals, without other opportunities for engagement. Similarly, another study described the activity of a CoP to support physical rehabilitation for people living with HIV as consisting of eight seminars over a 2‐year time frame [38]. However, this study also describes the process required to organise these seminars, which involved people living with HIV regularly coming together for planning sessions. These organisational activities were not described as being part of the CoP. The authors reported that the seminars were successful in maintaining participants over the 2‐year period, and that they championed lived experience and patient‐centred storytelling. Elsey and Lathlean [37] held monthly meetings over a period of 7 months, which they described as multisectoral debating forums. Through these, they reported that participants were able to draw on varied knowledge sources, including storytelling and experiences, to establish a common discourse. The authors also highlighted the importance of facilitators in ensuring that the voices of all participants (including lived experience experts) were heard and valued.

Other CoPs used a range of activities to create a shared repertoire. Giebel et al. [22, 23] described a combination of monthly, bimonthly, biannual and annual online and in‐person activities. These resulted in co‐production of research, grant applications and co‐production of a board game with people with lived experience who were members of the CoP. Muscat et al. [41] used a combination of an online hub, a mailing list, meetings, consultations, targeted training and a bimonthly seminar series formulated by participants, including those with lived experience. The CoP was described as being a central component of their co‐creation model, benefitting from the ability to reach a range of people with lived experience, resulting in many completed and ongoing research projects. Oliver et al. [20] similarly proposed using a combination of online meetings, webinars and virtual learning sessions. In Ekberg et al. [36], people with lived experience identified several important elements for the design of a CoP for adolescents with diabetes. These included having a variety of methods for interactivity between participants, such as discussion groups, conference systems and emails, as well as learning opportunities and support.

Other studies that similarly designed or investigated a CoP reported that it was an effective way to work with people with lived experience [47], offered a safe space for those with lived experience to connect and share [40], led to improved relationships and mutual learning between patients and healthcare professionals [34], and provided a valuable co‐creation process for participants [42, 45].

In contrast, the involvement of those with lived experience was not clear in one study. Johnson, Barach and Vernooij‐Dassen [39] described creating a CoP that included a group of researchers and patient advocates. However, throughout the study, the patient advocates are not explicitly referred to, making it unclear to what extent they were involved in the CoP activities.

### 3.7 Living Labs

Living labs were described in a number of ways in the included papers. Some referred to the ENoLL definition, describing living labs as user‐centred open innovation ecosystems [15, 18, 28, 48, 49, 50, 51, 52, 53], while others referred to the definition and key principles from Bergvall‐Kåreborn et al. [17] [29, 50, 54, 55, 56, 57, 58]. Several other sources were cited, with the included studies defining living labs as an approach for centring users in the co‐creation process for meaningful innovation, allowing for collaboration with diverse interest groups [24, 32, 52, 59, 60, 61, 62, 63, 64, 65, 66, 67, 68] and fostering communication between users and designers, which promotes user acceptance [26, 27, 63]. Living labs were described as involving technology installations in living environments and naturalistic settings [31, 63, 66, 67, 69, 70]. Palmer et al. [71] referred to the ENoLL definition; however, they chose to expand on it and operationalise the ENoLL concepts within a lived experience context, focusing on the importance of lived experience in medical and health research, and social innovation. One paper did not provide a definition of living labs [72].

### 3.8 Aim of Living Lab Studies

The living labs in the included studies focused mainly on the design, development and/or evaluation of products, services and practices, including technological and healthcare innovations. For some of the included studies, the aim of the article was the outcome of the living lab itself [52, 58, 59, 61, 62, 65, 66, 73, 74, 75], while other studies focused on the living lab methodology used for the development or evaluation process. This included developing living lab protocols to improve involvement of people with lived experience, for example, people living with dementia [50, 55, 56, 67], adolescents and young adults [32, 76], and older adults [24, 26, 27, 48, 57, 63, 77, 78, 79]. Studies also aimed to better understand the benefits and challenges of using a living lab [64, 80], including the transition and scalability to everyday use outside of the lab setting [69], the roles of those living and working in a living lab [25, 70], and understanding participatory practices and design in a living lab context [30, 54]. One study looked at using living labs in a novel participatory design methodology, which utilised aspects of business development [72]. Additionally, studies also explored different types of living labs, including a design‐driven approach [60], an Ambient Assisted Living Lab [31], a Digital Health Living Lab [51], a Public Intelligence Living Lab [81], a Living Lab Micro Innovation Cell [82], and eLabEL [28, 29]. These different types of living labs reiterate that there is not one interpretation of the living lab approach.

To a lesser extent, the living lab studies described networks for research. These were a network of healthcare services and community groups with a goal of promoting falls prevention [49], networks of long‐term care facilities for older adults with the goal of embedding scientific research [53, 68], and a co‐design collaboration driving research design to translation in mental healthcare [71].

### 3.9 Purpose of Using a Living Lab Approach

The rationale for using a living lab approach for the included studies was as a way to bring together different interest groups, for example, researchers and industry representatives with those with lived experience, to work together for a purpose which was most commonly to develop new technology or services [25, 26, 27, 28, 29, 31, 48, 50, 55, 56, 57, 58, 61, 63, 64, 65, 66, 67, 70, 72, 76, 78, 80, 81, 82], but also for research collaboration [49, 52, 53, 68, 71]. A living lab was also used because it offers an approach for centring, understanding and valuing the needs and opinions of end users [24, 26, 27, 29, 32, 48, 51, 52, 54, 55, 56, 57, 58, 60, 61, 64, 65, 66, 68, 71, 74, 75, 76, 77, 78, 79, 80, 81] and provides a methodology for testing products and services in a realistic setting [26, 27, 29, 31, 48, 50, 51, 55, 56, 57, 58, 60, 63, 64, 65, 67, 68, 73, 74, 75].

Archibald et al. [59] similarly used a living lab for co‐creation and valuing involvement of family members, but for a different purpose. A web‐based living lab platform was used for the collection of storytelling data from participants with lived experience and then to host these stories for the purpose of knowledge translation.

### 3.10 Settings and Methodologies of Living Labs

Living labs often took place in physical spaces, including participants' homes [50, 55, 57, 62, 63, 64, 67, 69, 74, 75, 79, 81]; nursing homes, care homes or retirement housing [30, 31, 48, 51, 60, 69, 70, 78, 80, 81]; and healthcare facilities [26, 27, 28, 29, 49, 57, 58, 65, 73, 77], providing naturalistic spaces for testing products or services. Other studies, particularly those focused on co‐design/creation, were often not defined by a setting, instead using the living lab approach as a framework for co‐design activities [24, 25, 32, 52, 53, 54, 56, 59, 61, 66, 68, 71, 72, 76, 82].

Prior to the testing phase, establishing or prioritising the needs of participants was an initial step for many studies. This was done using a Likert scale for needs ranking [54], workshops, focus groups or interviews [26, 27, 28, 29, 48, 51, 74, 79] and ethnographic studies [80]. The Senior Living Lab used bottom‐up techniques for investigating the needs of older adults and encompassed a number of different methodologies, including focus groups, World Café and ethnography [24, 25]. Pino et al. [57] describe tailoring research methods for older adults with cognitive disorders to determine their needs in less traditional ways.

Focus groups, workshops and interviews were used widely among the included studies not only for needs assessment, but also for generating ideas from participants [27, 32, 61], as a co‐design/creation method [51, 52, 56, 66, 72, 75, 76, 79, 82] and for evaluation of products or services [31, 50, 59, 61, 74, 77]. Specific co‐design activities used in focus groups and workshops were not always specified. Those which were described included live‐prototyping sessions, card sorting, a day in the life, emotion maps and animated scenarios [65, 71, 79].

### 3.11 Involvement of People With Lived Experience in Living Labs

The number or proportion of participants with lived experience was not always described. In papers where an exact number was stated, the number of participants ranged from 4 to 432, with a median of 14 [26, 28, 30, 32, 55, 58, 59, 60, 61, 62, 64, 65, 66, 72, 73, 74, 76, 77, 78, 79, 80, 81].

Several studies provided approximate numbers or ranges of participants. Palmer et al. [71] used the living lab approach to develop a network resulting in membership of nearly 2000 people, Verbeek et al. [68] covered 110 long‐term facilities with approximately 30,000 clients and more than 15,000 staff, and Vermeulen et al. [29] established living labs in primary care centres, resulting in recruitment of seven primary care centres with patient populations ranging from 3500 to 13,000 and a representative patient panel of 250 members at each centre. Furthermore, one study referred to the number of households in the living lab (*n* = 258) [81] and another stated the number of care homes (*n* = 10) [70]; neither detailed the number of individuals in these settings.

People with lived experience were often involved throughout the study in living labs that used co‐design or participatory design methods. This typically included participation in needs assessments, co‐design workshops and idea incubators before product development and testing [24, 25, 27, 29, 32, 51, 52, 54, 56, 59, 61, 64, 65, 66, 71, 72, 74, 75, 76, 77, 78, 81]. Palmer et al. [71] and Swist et al. [32] were examples of comprehensive lived experience involvement, demonstrated by continuous engagement, co‐learning and co‐leadership, remuneration, informal mentoring, referring to people with lived experience as co‐designers or commissioners, and providing opportunities for co‐authoring and co‐research. Verbeek et al. [68] similarly included people with lived experience as collaborating partners and described continuous dialogue for identifying research themes. Gimenez et al. [52] described working with stakeholders to determine the research needs, followed by the creation of three living labs, each with its own agenda, governance and cocreation processes that were co‐led and co‐created by the stakeholders, including migrant communities, patients' associations and the scientific community. Three studies also involved advisory groups of people with lived experience who informed research or development of the outcome [53, 54, 59].

Some studies described approaches for inclusion of people with lived experience in the living lab process, including user‐centred, inclusive design methodology [48], cyclical participatory design process [61], and the Co‐Design Living Labs Model [71]. For some co‐design/creation studies, specific methods were not always described, leading to difficulty in understanding how participants were involved in the process [61, 63, 69, 72]. Additionally, it was not always clear where the living lab approach began and ended. Some studies included users in a co‐design process, but often this part of the methodology was described as occurring before the living lab had begun [55, 69, 73].

Some studies did not adequately report on the inclusion of lived experience experts or only involved them in product evaluation and feedback sessions [31, 62, 67, 82]. In two articles by Brankaert and Ouden [50, 60], the use of living labs for co‐creation is described; however, it seems that lived experience experts evaluated a product but were not involved in the development process of the product. In Blain et al. [49], those with lived experience were only represented through patient organisations, despite reference to user‐driven open innovation. One study intended to involve lived experience experts but had difficulty in recruitment [28] (Table 2).

**Table 2:** Summary of elements of CoP and living labs that involved people with lived experience identified in this review.

	Community of practice	Living lab
Aim of the included studies	Support systems for people with lived experience. Improvement of health literacy, services and policies.	Co‐design, development and evaluation of products and services. Collaborative networks.
Purpose of use of the approach	Knowledge exchange, knowledge to action, network building.	Collaboration between academia, industry and end users; valuing the input and needs of end users; ability to test products/services in realistic settings.
Key study design and methodologies	Participatory design or action research. Web‐based platforms, group discussions, webinars.	Co‐design, co‐creation or participatory design. Focus groups, workshops and interviews.
Setting	Online and in person.	Mostly in person.
Involvement of people with lived experience	Varied. CoPs often formed part of co‐design processes. Lived experience expert involvement in the whole research process was limited.	Varied. Living labs frequently form part of a co‐approach process. There was some lived experience expert involvement across the whole research process.

## 4 Discussion

CoPs and living labs are two frequently employed approaches for involving people with lived experience in research. This scoping review examined the key characteristics of CoPs and living labs in the health and healthcare space that involve people with lived experience to understand their key characteristics and how they compare.

CoPs and living labs were utilised for different purposes, as indicated by their definitions. CoPs are built around collective learning and thus were most often used for knowledge exchange and building networks [11, 12]. Living labs, on the other hand, are defined as innovation ecosystems for co‐design, and thus were mainly used in the design and evaluation of products and services [15]. This was similarly reflected by the involvement of industry partners [28, 49, 51, 55, 60, 67, 70], compared to CoPs, which were largely initiated by researchers or participant needs.

In some cases, living labs also referred to research networks, and it was in these instances that both approaches shared similarities. Verbeek et al. [68] presented a living lab model for collaboration between researchers and end‐users, including older persons and their families. The living lab is described as not being a physical space, but rather a network that drives scientific research through continuous collaboration and discussion with end users in long‐term care. This interpretation aligns with the knowledge exchange and network‐building elements identified in CoPs.

Similarly, Palmer et al. [71] describe a co‐design living lab which aims to drive research innovation and translation in mental healthcare and research. The living lab has grown to include 2000 participants who help to identify research priorities and shape research questions and approaches and has some people with lived experience in co‐researcher roles. The paper describes lived experience as a form of knowing, an element that is discussed in CoP literature. This living lab has similar goals to the previously described CoP, the Liverpool Dementia & Ageing Research Forum [22, 23]. These studies differ in the methodologies used; however, Palmer et al. [71] describe co‐design methods, including A Day in the Life, Emotion Maps and Card Sorting, while Giebel et al. [22, 23] utilise webinars, networking meetings and conferences. Similarly, Riva‐Mossman et al. [25] and Angelini et al. [24] describe the Senior Living Lab as a *‘social learning space fostering encounters among colearners’* [25]. This definition draws on similar concepts to a CoP, and the papers subsequently reference Wenger's work on social learning systems.

Furthermore, two additional living labs also used a different interpretation of the common ENoLL definition for the approach. Monguet et al. [82] used a virtual platform to present ideas to participants, host discussion forums and video conferences and manage the co‐creation of innovative eHealth services. The paper did not describe user testing of the innovations, and as such, it is difficult to differentiate the approach from that of CoP articles, which similarly developed policies and materials for healthcare improvement. Archibald et al. [59] described the living lab as infrastructure and a collaborative space. The living lab was used for data collection and then served as a platform for knowledge exchange, due to its capabilities for hosting co‐design, enabling cost‐effective feedback cycles. The creation of a knowledge exchange platform was similar to the purpose of the included CoP articles.

As described by Bergvall‐Kåreborn et al. [17], living labs can be interpreted in a number of ways, which was the case in the included studies. Whereas the application of CoPs tended to be more consistent across the included studies. The broader interpretations of living labs resulted in convergence of the two approaches in some instances; however, living labs in these convergent cases typically referred to large‐scale networks or organisations, while CoPs referred to groups of individuals.

### 4.1 Strengths and Limitations

This review had some limitations. A quality appraisal of the articles was not performed, as the purpose of this review was exploratory in nature. Critical appraisal is not mandatory within the current PRISMA‐ScR reporting guidelines [19]. Articles appropriate for inclusion may have been missed due to the large number of terms used to refer to the involvement of those with lived experience [6]. However, a range of different terms were used in our search strategy, which did result in many articles from a range of disciplines. The search was limited to English‐language articles for pragmatic reasons, which may have biased the sample to those published in Western contexts and potentially omitted studies published in other languages. There was difficulty in extracting some data from the included studies due to variability in reporting. Some studies did not adequately report the specific methodologies, settings or participant numbers, which limited the ability to interpret and compare the results. This has similarly been reported elsewhere for both living labs and CoPs, with reviews finding that many studies were inconsistent in their reporting or were presented without a rigid methodology [83, 84]. This review also did not conduct exhaustive searching of the reference lists of each included study beyond the reviews which were identified in the abstract screening.

This review also had some strengths. This is the first time a review has been undertaken comparing and contrasting the key elements of living labs and CoPs that include people with lived experience to understand how they are being used in health and healthcare research. As such, a tailored extraction template was used to understand these key elements.

## 5 Conclusion

Living labs and CoPs are effective ways of including people with lived experience in the research process. Their differing purposes make them appropriate for different contexts, but there is flexibility in how the approaches can be applied. Typically, CoPs are used for instances of creating and sharing knowledge between individuals, while living labs are utilised for the creation and testing of technology, or for large‐scale research networks. CoPs and living labs both offer a range of benefits, as inclusion of people with lived experience is crucial in health and healthcare research, and a requirement in some countries such as the United Kingdom.

## Author Contributions

**Eliza Watson:** investigation, formal analysis, project administration, methodology, visualisation, writing – original draft, writing – review and editing. **Sadia Afrin:** investigation, writing – review and editing. **Katrina M. Long:** methodology, validation, writing – review and editing. **Clarissa Giebel:** funding acquisition, writing – review and editing. **Chris Moran:** funding acquisition, writing – review and editing. **Darshini Ayton:** conceptualisation, funding acquisition, methodology, validation, supervision, writing – original draft, writing – review and editing.

## Disclosure

The views expressed in this publication are those of the author(s) and not necessarily those of the National Institute for Health and Care Research or the Department of Health and Social Care.

## Ethics Statement

The authors have nothing to report.

## Conflicts of Interest

The authors declare no conflicts of interest.

## Supporting information

Supplemental file 1.

Supplemental file 2.

Supplemental file 3.

## Data Availability

Data is available upon reasonable request to the corresponding author.
