# Supplementary material for: Communities of Practice and Living Labs: A Scoping Review of Principles and Methodologies for Involvement of Lived Experience Experts in Health and Healthcare Research
Source: Health Expect. 2026 Feb 22;29(1):e70601. doi: 10.1111/hex.70601 (PMC12928026; doi:10.1111/hex.70601)
Supplement: Supplementary file 1 — Supplemental file 1. [file HEX-29-e70601-s003.docx]

**Supplemental File 1. Search strategy**

**Medline**

(“living lab*” OR “community of practice”) AND (“patient and public involvement” OR “consumer and community involvement” OR “public and patient involvement” OR “Community Participation/” OR “community participation” OR “Patient Participation/” OR “patient participation” OR “academic-practice partnership” OR “Community-Based Participatory Research/” OR “research partnership” OR “Community-Institutional Relations/” OR “experience co-design” OR “co-design” OR “multi-stakeholder collaboration” OR “co-creation” OR “co-creat*” OR “participatory design” OR “lived-experience”)

**Scopus**

( ( TITLE-ABS-KEY ( "consumer and community involvement" ) ) OR ( TITLE-ABS-KEY ( "patient and public involvement" ) ) OR ( TITLE-ABS-KEY ( "lived experience" ) ) OR ( TITLE-ABS-KEY ( {lived experience} ) ) OR ( TITLE-ABS-KEY ( {lived-experience} ) ) OR ( TITLE-ABS-KEY ( "academic-practice partnership" ) ) OR ( TITLE-ABS-KEY ( "research partnership" ) ) OR ( TITLE-ABS-KEY ( "experience co-design" ) ) OR ( TITLE-ABS-KEY ( "multi-stakeholder collaboration" ) ) OR ( TITLE-ABS-KEY ( "co-creation" ) ) OR ( TITLE-ABS-KEY ( "participatory design" ) ) OR ( TITLE-ABS-KEY ( "patient participation" ) ) OR ( TITLE-ABS-KEY ( "community participation" ) ) ) AND ( ( TITLE-ABS-KEY ( "living lab" ) ) OR ( TITLE-ABS-KEY ( {living lab} ) ) OR ( TITLE-ABS-KEY ( "community of practice" ) ) OR ( TITLE-ABS-KEY ( {community of practice} ) ) )

**CINAHL**

(“living lab*” OR “community of practice”) AND (“patient and public involvement” OR “public and patient involvement” OR “consumer and community involvement” OR (“community participation” OR “community engagement” OR “community empowerment”) OR (MM “Patient Participation”) OR (“patient participation” OR “patient involvement” OR “patient empowerment” OR “patient engagement”) OR (MM “Consumer Participation) OR “consumer participation” OR “community based participatory research” OR (MM “Community-Institutional Relations”) OR “community-institutional relations*” OR “experience co-design” OR “co-design” OR (MM “Stakeholder Participation”) OR “multi-stakeholder collaboration” OR “co-creation” OR “participatory design” OR (“lived experience” OR “lived-experience”))
