# Supplementary material for: Communities of Practice and Living Labs: A Scoping Review of Principles and Methodologies for Involvement of Lived Experience Experts in Health and Healthcare Research
Source: Health Expect. 2026 Feb 22;29(1):e70601. doi: 10.1111/hex.70601 (PMC12928026; doi:10.1111/hex.70601)
Supplement: Supplementary file 3 — Supplemental file 3. [file HEX-29-e70601-s001.docx]

**Supplemental file 3 – Custom Data Extraction Template**

- Author/s
- Title
- Year
- Country in which the study was conducted
- Type of study (Community of Practice (CoP) or Living Lab)
- Aim of study
- Context of the study
  - If not covered by the aim or context, what was the goal of the CoP or Living Lab?*
- Stated purpose of using the CoP or Living Lab approach
- Setting
- Was a definition of a CoP or Living Lab provided?
  - If yes, where was this definition taken from?
  - How was it defined?
- What components or activities were used in the CoP or Living Lab?
- What other methodologies were used if the study had more stages outside of the CoP or Living Lab?
- Participant description
  - Inclusion criteria
  - Exclusion criteria
  - Total number of participants
- If evaluation was conducted, what was the authors’ and participants’ experience of using a CoP or Living Lab?

*For some studies, the aim of the paper differed from the goal of the CoP or Living Lab. It was important to capture both aspects.
